# Urban conservation hotspots: predation release allows the grassland-specialist burrowing owl to perform better in the city

**DOI:** 10.1038/s41598-017-03853-z

**Published:** 2017-06-14

**Authors:** Natalia Rebolo-Ifrán, José L. Tella, Martina Carrete

**Affiliations:** 1 Departamento de Ecología, Genética y Evolución & IEGEBA-CONICET, Facultad de Ciencias Exactas y Naturales, Universidad de Buenos Aires, Buenos Aires, Argentina; 2 Grupo de Investigadores en Biología de la Conservación (GRINBIC) INIBIOMA-CONICET, Bariloche, Argentina; 3 Department of Conservation Biology, Estación Biológica de Doñana, CSIC, Sevilla, Spain; 4 Department of Physical, Chemical and Natural Systems, Universidad Pablo de Olavide, Sevilla, Spain

**Keywords:** Biodiversity, Conservation biology, Urban ecology

## Abstract

Although habitat transformation is one of the main causes of biodiversity loss, there are many examples of species successfully occupying and even proliferating in highly human-modified habitats such are the cities. Thus, there is an increasing interest in understanding the drivers favoring urban life for some species. Here, we show how the low richness and abundance of predators in urban areas may explain changes in the habitat selection pattern of a grassland specialist species, the burrowing owl *Athene cunicularia*, toward urban habitats. Predation release improves the demographic parameters of urban individuals, thus favoring an increment in the breeding density of the species in urban areas that accounts for the apparent positive selection of this habitat in detriment of the more natural ones that are avoided. These results suggest that traditional habitat selection analyses do not necessarily describe habitat choice decisions actively taken by individuals but differences in their demographic prospects. Moreover, they also highlight that cites, as predator-free refuges, can become key conservation hotspots for some species dependent on threatened habitats such as the temperate grasslands of South America.

## Introduction

Humans have transformed ecosystem patterns and processes across the biosphere, with a critical terrestrial transition from mostly wild to mostly anthropogenic between 1,700 and 2,000^1^ that precipitated a major global biodiversity crisis^2^. However, not all species respond equally to habitat transformations, and there are many examples of species occupying and even proliferating in transformed environments^3, 4^. In this sense, there is an increasing interest in studying how species colonize and thrive in novel habitats^5, 6^ as well as understanding the drivers of change in habitat preferences shown by many species as a response to anthropogenic habitat changes.

Changes in habitat selection processes may be involved in the preferential use or avoidance of new anthropogenic habitats. Habitat selection is defined as the process by which an animal chooses between habitats and is usually measured by the use of a particular habitat relative to its availability^7, 8^. This last point is important, as habitat availability can affect the strength of habitat selection, so when a habitat becomes rare, selection could be stronger compared to when it is common^9, 10^. For species that may utilize a wide range of habitats, quantifying habitat selection is particularly challenging, spatial and temporal changes in resource abundance or ecological pressures can result in changes in selection preference affecting the robustness of habitat selection models. Thus, understanding the complex, dynamic interaction between species occurrence and habitat patterns is essential to predict and manage responses of species to natural or anthropogenic environmental changes.

Besides critical resources such as food and breeding sites^7, 11^, predation risk may be an important determinant during the habitat selection process^12–14^. Presence and abundance of predators can change across habitat types^15^ so that variability in predation risk creates heterogeneous “landscapes of fear” that could be important during the habitat selection process^16^. In this sense, because predators tend to be more sensitive than prey to habitat changes and human disturbances, some species might benefit (via release from predation) from habitat transformations, thus positively selecting human-modified environments^17–20^.

In this paper, we investigated breeding habitat selection in the burrowing owl (*Athene cunicularia*), a species typically associated to North and South American grasslands that can also breed in habitats with different degrees of transformation, including cities^21–23^. In particular, we were interested in understanding differences in the occupation of urban and rural habitats. Therefore, we assessed whether the abundance of breeding pairs in each habitat was proportional to its availability or, conversely, if habitats are differentially occupied, through a habitat selection approach. As a potential driver of habitat selection, we evaluated differences in predator richness and abundance between both habitat types. Our results show a positive selection of urban sites over the much more extended natural and rural habitats. The abundance and richness of predators was much lower in urban areas, which can explain the higher demographic parameters and breeding densities of burrowing owls in the city. These results suggest that predation pressure through top-down regulatory processes could be one important driver of the population bloom of some avian urban populations. Additionally, they highlight the role of cities as conservation hotspots for some species that are now thriving better in urban areas than in their increasingly threatened natural habitats.

## Results

### Distribution of breeding pairs between habitats and breeding densities

**Figure 1:**
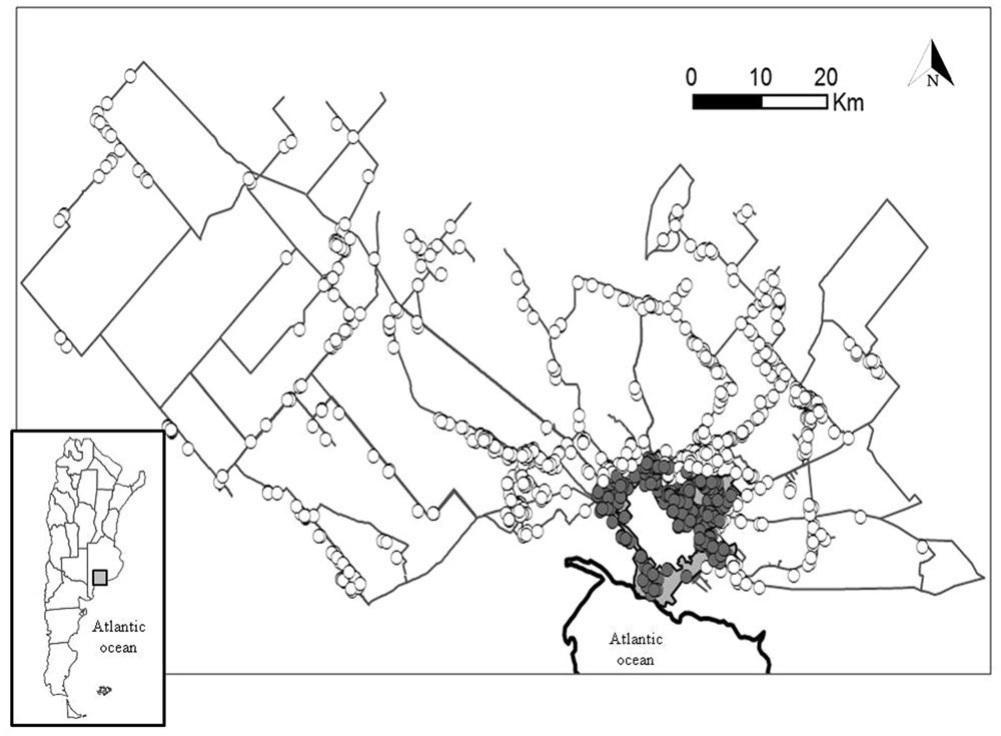
Study area showing urban and rural nests (grey and white dots, respectively) of burrowing owls. Grey lines represent transects used to assess availability of rural habitats. Note that many dots overlap. Map was generated with Q-GIS 2.8.1 (http://qgis.osgeo.org).

We recorded 1,749 active nests of burrowing owls distributed across the years (2008–2013) and habitats present in the study area (Fig. 1). Most of the rural nests were located in grazed habitat plots (grazed grasslands and regenerating grazed grasslands, n = 273 and 201, respectively), being scarcer in more natural areas (natural grasslands and regenerating grasslands, n = 94 and 86, respectively) and crops (n = 61). The rural area represented on average 81% of the total surveyed area (322.77 km^2^), and was mainly covered by natural grasslands (41%) and crops (21%), with less relevance of the other habitat types (<14%). On the other hand, 432 nests were located in the urban area during the study period (Fig. 1) over a surface of 74 km^2^ (29% of the total surveyed area).

**Figure 2:**
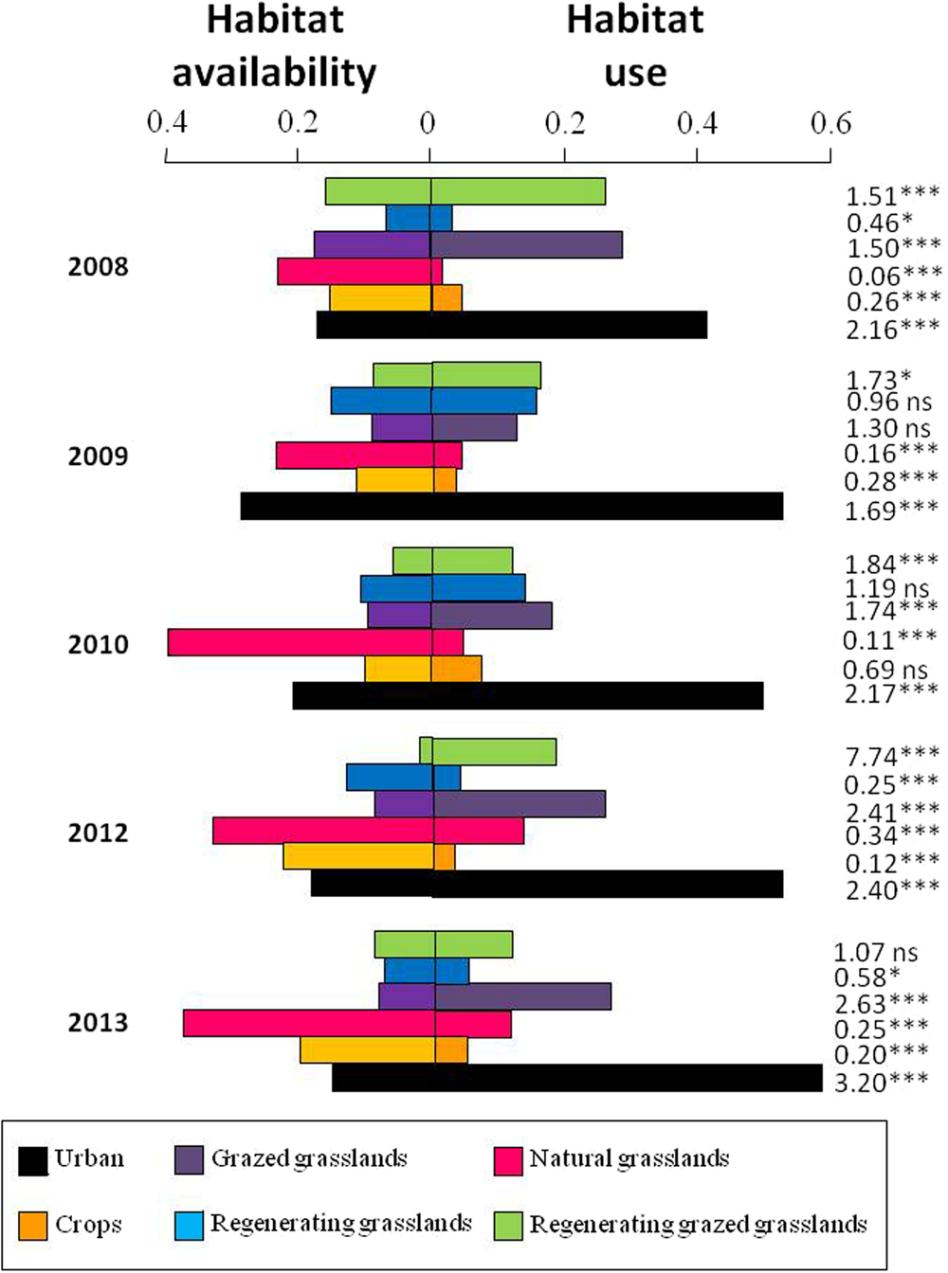
Availability and use of different habitats by burrowing owls. Savage selectivity indexes are shown right to the bars (***p < 0.0001; **p < 0.001; *p < 0.01; ns: p > 0.05).

**Figure 3:**
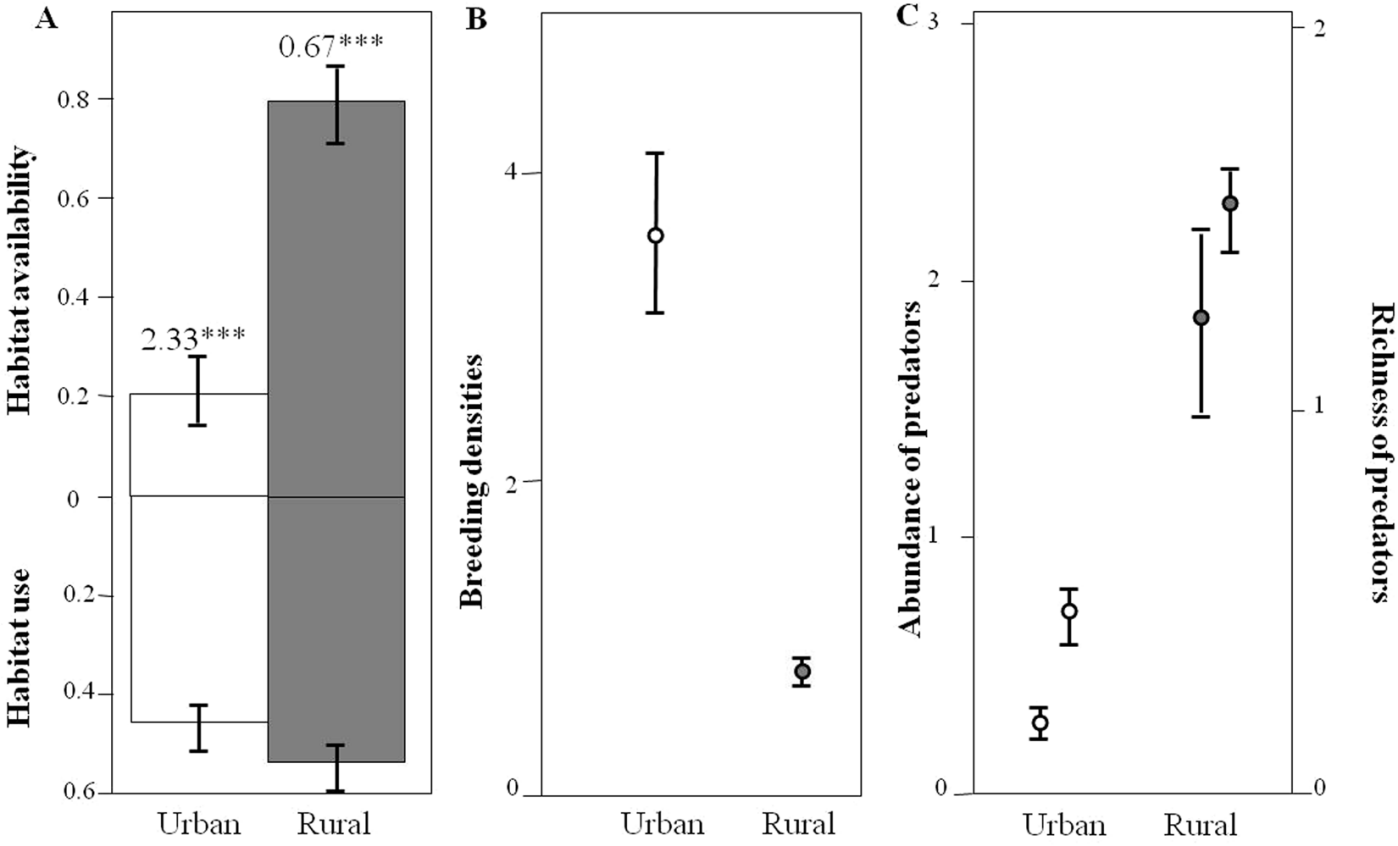
(**A**) Habitat availability and use, (**B**) breeding densities (number of breeding pairs/km^2^) of burrowing owls and (**C**) abundance (individuals/h) and richness (species/h) of potential predators in urban and rural habitats. Savage selectivity indexes are shown above the bars in A (***p < 0.0001). Dots represent mean values while bars depict SE.

Savage selectivity indexes indicated that burrowing owls negatively selected natural grasslands and crops and positively selected grazed grasslands, with other rural habitats showing less consistent patterns across years (Fig. 2). Overall, owls preferentially used urban over rural habitats (Savage selectivity indexes: *W*_urban_ = 2.46; *W*_rural_ = 0.67; Fig. 3A), a pattern that remained highly consistent along years (Figure S1). Accordingly, urban landscapes showed average densities 7 times higher than rural ones (rural density: 0.52 ± 0.05 breeding pairs/km^2^; urban density: 3.64 ± 0.83 breeding pairs/km^2^, Fig. 3B).

### Abundance and richness of predators

We recorded 1,137 potential predators of burrowing owls during 2,073 h of fieldwork, 966 predators during 1,158 h in the rural areas and 171 predators during 915 h in the urban areas. The abundance of predators differed highly between urban and rural landscapes (Table S1).

The average abundance of predators was near six times higher in rural areas (1.84 ± 0.37 indiv/h) compared to urban habitats (0.27 ± 0.05 indiv/h, F = 157.94, p < 0.0001; Fig. 3C). Predator richness was also higher in the rural areas (1.50 ± 0.07 species/h) than in the urban areas (0.46 ± 0.04 species/h; χ^2^ = 117.96, p < 0.001; Fig. 3C). In both cases, the duration of the fieldwork session and year of sampling were retained in models (all p < 0.0145). This pattern remained constant when both terrestrial and aerial predators were separately analyzed (Table S2).

### Breeding performance

We monitored 3,161 breeding events from 2007 to 2014 in both rural (n = 1,813) and urban (n = 1,348) habitats. Breeding failure affected 27% of the nests, being higher in rural habitats (45%) than in urban ones (27%, Yate’s corrected χ^2^: p < 0.0001). Although some nests were lost after intense rain, house building or land management, predation was the main known cause of breeding failure both in urban (83%, n = 64) and rural nests (80%, n = 117). Extrapolating these proportions to the entire urban and rural unsuccessful nests, we estimated that 22% of urban and 36% of rural territories were predated (Yate’s corrected χ^2^: p < 0.0001).

Differential predation risk among habitats seemed to translate into higher reproductive parameters in urban rather than in rural pairs (breeding success: urban: 73%, rural: 55%; χ^2^ = 122.98, p < 0.0001; brood size: urban: 2.85 ± 0.04; rural: 2.76 ± 0.04; F = 4.70, p = 0.0303; productivity: urban: 2.08 ± 0.04; rural: 1.50 ± 0.04; χ^2^ = 79.56, p < 0.0001), after controlling for inter-annual variability in all breeding parameters (all p < 0.05).

## Discussion

Burrowing owls are open area specialists, foraging and breeding in short, low-density vegetation, mainly grasslands. As this is one of the most endangered habitats of the world due to agricultural and urban development, many burrowing owl populations are declining, and the species is protected in USA, Canada and Mexico^24, 25^. Our results, however, suggest that some burrowing owl populations are able to exploit human-made habitats, as shown by the burrowing owl’s preference of urban over rural areas (natural grasslands and low-intensive agro-pastoral lands) consistently shown in our study area over years. Differences in predation risk, as measured by the abundance and richness of predators, may explain why breeding density was seven times higher in urban than in rural areas. A habitat selection pattern related to predator risk was also detected within the rural habitats, where owls consistently avoided the most natural habitats, selecting areas highly used for livestock grazing. These results are in agreement with other studies where owls select habitats with short, sparse vegetation, avoiding habitats with high, dense pastures^26^. Livestock grazing keeps the vegetation short, creating an open habitat that can allow for early detection of predators as traditionally did fossorial mammals such as prairie dogs (*Cynomis sp*) or plain viscachas (*Lagostomus maximus*) in North and South America, respectively. Overall, predation may act as the main ecological force driving an unexpected pattern of habitat selection, where burrowing owls seem to prefer the most human-modified habitats (urban > grazed grasslands > natural grasslands).

Habitat selection is usually considered as a consequence of animals actively selecting where to live, reflecting behaviours mechanistically linked to movement^27^. However, abiotic and biotic processes can create heterogeneity in the distribution of ecological conditions (e.g., microclimates, food, vegetation cover) that can translate into individual’s fitness^28, 29^, so apparent habitat selection patterns can passively arise as a consequence of differences in the ability of a species to persist or even proliferate in certain habitat types instead of in others. Predators can control prey populations by the lethal removal of individuals, creating a top-down regulatory mechanism commonly invoked to explain the composition and dynamics of natural populations^30, 31^. However, the actual impact of this consumptive effect on population abundances is controversial (e.g., refs 30–35), mainly because the heterogeneity in predation pressure across landscapes can affect its relative importance in front of other regulatory mechanisms such as bottom-up or parallel ones^16^. The “landscape-of-fear” model^36, 37^ has helped to understand these inconsistencies, showing that the proportion of risky to safe habitats across a landscape can affect the relative importance of regulatory mechanisms, both top-down but also bottom-up, in determining the absolute population density of a species^16^. Here, we show how predation can have direct impacts on rural birds by reducing their breeding success and, therefore, their productivity. Although also lower among rural birds, brood size was less affected, suggesting that the low reproductive performance in rural habitats is not because of differences in food resource availability, as seems to happen among some passerines^38^ but mainly as a consequence of predation that removes entire clutches or broods^39^. Previous work suggests that predation can also contribute to reducing survival rates of these individuals, as shown by the lower annual survival estimates obtained for rural compared with urban burrowing owls (φ_urban_ = 0.59; φ_rural_ = 0.25^40^). Altogether, higher survival and reproductive output in urban than in rural habitats paralleling differences in predation pressure suggest that the apparent switch of the species to prefer urban over natural habitats can arise as a passive consequence of the differential demography of urban and rural subpopulations rather than through active habitat selection processes at the individual level. This idea is supported by the short natal and breeding dispersal distances shown by these burrowing owls (medians 0.30 and 0.02 km respectively, Authors in prep.), so individuals living close to the city could prospect and freely choice among different rural and urban habitats, but the urban choice would be hard for individuals living farther (Fig. 1).

Although evidence is not conclusive^41–44^, urban areas have been considered as refuges from predators for wildlife^39, 45^. The “predator refuge hypothesis” states that urban species thrive because they are released from predation pressures that they would ordinarily face in the wild. However, people present in urban sites can be perceived as potential predators, regardless of whether or not they pose a genuine threat to animals^46–48^, thus contributing to increase the heterogeneity in the landscapes of fear^49, 50^. Fear of humans has been largely overlooked as a behavioural trait precluding the colonization of urban environments. However, recent work has shown that only tame individuals from species showing high intraspecific variability in fear of humans could colonize urban areas^22^. This results in significantly lower flight initiation distances among urban individuals compared with rural ones^22, 51^ without suffering the costs, measured as long-term stress, of living in close contact with humans^40^. Therefore, only species with individuals able to colonize and thrive in urban areas could benefit from the predator release effect through an improvement of their fitness parameters. More research is however needed to properly understand the ecological and evolutionary consequences of the different demographic dynamics between urban and rural populations of urban dwellers, and the roles played by dispersal as a potential force that homogenizes or, contrarily, reinforces the differences between them^52^.

### Cities as conservation hotspots for native species

By 2030, approximately 5 billion of the world’s 8 billion residents will live in urbanized areas, with projections of nearly 6 million square kilometers of land converted to urban areas. As both the number of people and the area of developed land on Earth increase, so does the potential for negative effects on biodiversity^2^. Threats to biodiversity come from direct land conversion and from increased colonization by introduced species, that result in a reduction on the number of native species present in urban areas. Although random processes account for part of this species loss, urbanization causes the simplification and homogenization of avian communities through extinctions^53^, with endemic and threatened species being especially affected^54^. However, in many cases, urbanization also leads to a reduction of predators^55^. Cites can thus emerge as predator-free refuges where species able to colonize urbanized habitats by individuals with heritable tolerance to humans^22, 40, 56^ can thrive and flourish. Larger population densities or abundances in urban than in rural habitats have been also recorded for other species^22, 45^, suggesting a significant role of predation release in the success of urban dwellers^39, 57^.

Cities can thus become key conservation hotspots for species that, unexpectedly as it is our case, now perform better there than in their currently threatened natural habitats. For example, Australian cities actually support substantially more nationally threatened animal and plant species than non-urban areas^58^ and 22% of the occurrences of endangered US plant populations are located in the 40 largest metropolitan areas^59^. Although in both cases authors argue that cities are relatively young and may be carrying extinction debts, our results support that, at least in some cases, this urban life can actually be the consequence of a successful occupation of this new habitat. Therefore, while urbanization should be considered a threatening factor for many species, it should be also viewed as critical conservation areas for others. In this sense, wildlife conservation should be integrated into urban planning through different strategies, such as the creation of green corridors or the maintenance of open spaces, depending on the species considered. The potential value of gardens for enhancing biodiversity has long been recognised^60^, and public ‘gardenwatch’ initiatives, such as the Big Garden Birdwatch promoted by Royal Society for the Protection of Birds (http://www.rspb.org.uk/birdwatch/) and the British Trust for Ornithology Garden BirdWatch (http://www.bto.org/gbw/) in the UK, and Project FeederWatch in the USA and Canada (http://www.birds.cornell.edu/pfw/), underline the importance of gardens for raising awareness about biodiversity and the public understanding of science. This ‘citizen science’ movement has huge potential for enhancing urban environments by coordinating public management actions to produce cumulative positive impacts on biodiversity^61^. However, a more effective ‘citizen urban conservation science’ can be boosted by fuelling people with scientific information showing which species have major conservation problems in nature^54^, thus requiring focused conservation efforts in cities, and which are the drivers of successful urban life for some species, as shown here, to manage them.

The relative role of predator release as a driver of successful urban life may vary between and even within cities, to the point that the rarefaction of predators may favor some species in some urban areas while local increases in predator numbers can lead to decreases in other urban populations of the same species through impacts on their survivorship or reproduction^62^. In some areas, mesopredators such as coyotes, foxes, and hawks are abundant in the surrounding of urban habitats. Others, including introduced and domestic species such as cats, increase proportionally with human density. For example, free-ranging domestic cats can kill 1.3–4.0 billion birds and 6.3–22.3 billion mammals annually in USA, un-owned cats causing the majority of this mortality^63^. However, it has been shown that some prey populations actually have greater survival rates in urban areas, and hypotheses proposed to explain this paradox are (1) the large quantities of food often available in cities to wildlife, (2) the hyper-abundance of co-existing prey populations in urban habitats, thus causing predator satiation, (3) the high specialization of predators on particular prey species, reducing the predation pressure on other prey populations, (4) the different predator species living in cities (species present in urban areas have a lower predator pressure in both urban and rural sites compared with species not present), which exert less pressure on prey populations^64, 65^, and (5) the prey composition that also changes in urban landscapes (so most of the species that remain in urban habitats are well-adapted to urban predators)^66^. In any case, efforts to reduce the impact of some predators of no conservation concern, such as domestic cat, can help to improve the prospects of urban biodiversity.

Although predation release is an important determinant of successful city life for some species in certain areas, other factors should be also taken into account to understand and preserve urban biodiversity. Recently, Beninde *et al*.^67^ suggest that increasing the area of habitat patches and creating a network of corridors is the most important strategy to maintain high levels of urban biodiversity. However, authors recognize that this result does not take into account the potential increments on invasive species, which presence has been shown to be negative for some native species of conservation concern^68^. Moreover, habitat improvements can also increase the presence of predators in cities, thus potentially increasing predation risk for some species. Thus, future research should assess the relative role of all factors involved in allowing urban life in different taxa. Importantly, these studies should be performed not using traditional richness or diversity indexes but focusing in the conservation ‘quality’ of species if we really want to give cities a true role in the conservation of biodiversity.

## Methods

### Study area and habitats

The study was carried out in an area of ca. 6.200 km^2^, comprising the city of Bahía Blanca and its surrounding rural areas (38°43′S 62°16′W; Buenos Aires, Argentina; Fig. 1). This relatively young city (founded in 1828) was a small village until the middle of the twentieth century, reaching 300,000 inhabitants in 2010. The city is located in the semi-arid Pampas, a large area consisting of natural grasslands interspersed in a matrix of transformed lands dedicated to low-intensive agriculture and livestock grazing. This ecoregion is actually considered as endangered and is regarded as a high priority conservation area at the regional scale^69^.

Some burrowing owls occupy the residential areas surrounding the core of the city. There, urban pairs excavate their own nests in private and public gardens, spaces between houses, street curbs and along large avenues, usually within 10–100 m of inhabited buildings^23^. Rural pairs breed in the surrounding expanses of natural grasslands and pastures devoted to wide-ranging livestock and low-intensive cereal crops, where owls also excavate their own nests but can also occupy burrows from fossorial mammals^70^. The city is immediately surrounded by large and flat rural extensions, with no clear interface between urban and rural habitats^51^. Unpaved roads cross these rural areas with very low pedestrian and vehicle traffic^22^, which allow farmers and rangers to access their low-intensive managed lands. These private lands are fenced to avoid the free movement of livestock. Therefore the rural landscape is composed of a mosaic of squared fenced plots (averaging 1 km^2^) that are subject to different management regimes, namely: (1) natural grasslands (grasslands approaching the pristine ecosystem of the region), (2) grazed grasslands (natural grasslands exposed to livestock grazing), (3) active crops (mostly wheat), (4) regenerating grasslands (abandoned crops with advanced regeneration of grasslands), and (5) regenerating grazed grasslands (as above, but grazed by livestock). Management of these habitat plots rotates and thus their relative surface varies across years. Other natural habitats (dry forests and scrubs, and salt lakes) are very marginal and are never used for breeding by burrowing owls. Burrowing owls have small home-ranges, with most of the activity restricted to the surroundings of their nests along the annual cycle. Although we have no detailed data on movements obtained from radio-tagged individuals, our observations of hundreds of banded individuals across ten years^23, 40, 51, 56^ indicate that birds stay most of the time close to the nests during both the breeding season (from the mating to the chick fledgling stages), for mating, resting, hunting, guarding and raising young, moving away only for short periods of time that restrict the possibility of performing large movements, and the non-breeding season. Accordingly, radio-tracked burrowing owls in Florida, where the population of the species is also sedentary, show that most of the movements of the individuals took place in the immediate surroundings of the nests, typically within a radius of 50 m^71^. Therefore, even when we actually measured breeding habitat selection, our study model is not similar to the situation observed in other raptors daily moving up to tens of kilometres for hunting during the breeding season or thousands of kilometres for reaching their breeding quarters, and thus our approach can be considered as an integrate habitat selection study.

### Location of breeding pairs

The study area was repeatedly visited –almost daily– across six breeding seasons (November-January of 2008–2013) to locate breeding pairs and geo-reference their active nests. Nests are easily located since breeders show diurnal activity and usually perch in the entrance of their burrows or on nearby small bushes and fences.

The discrete extension of urban areas allowed us to yearly conduct a complete census of the breeding pairs by driving a car through the streets and on foot. Censusing the whole surrounding rural area was however logistically impractical, so we relied on the survey of a number of unpaved roads, distributed across rural areas to cover the whole spatial variability (Fig. 1), to locate breeding pairs and estimate breeding densities, as well as habitat use and availability. We repeatedly drove a car at low speeds (20–30 km/h) through the selected roads, with a driver and at least one observer (e.g. ref. 15), and stopped every time we observed a new owl in order to locate its nest.

### Habitat availability

Habitat availability slightly changes annually and thus was separately evaluated for each breeding season from 2008 to 2013, with the exception of 2011 due to logistic constraints. Habitat availability in rural areas was estimated by recording the number of each habitat patch on both sides of the unpaved roads. Thus, we measured habitat availability in real time and at the same spatial scale in which habitat use is estimated, assuming that, in large sample sizes, plot size approaches the mean size of all plots evaluated^72, 73^. An average plot length (i.e., the length of the plot in contact with the road) was estimated by measuring it in 60 randomly selected fenced plots across the study area using aerial photographs. Plot width (i.e., perpendicular distance from the road) was estimated using the distance *D* within which we located 99% of the nests, that varied among habitats depending on vegetation cover and height (natural grasslands: 628 m, grazed grasslands: 618 m, crops: 468 m, regenerating grasslands: 252 m, regenerating grazed grasslands: 664 m). The total availability of rural habitat was therefore obtained as $\Sigma \bar{L}\ast {n}_{i}\ast {D}_{i}$, where *n*_*i*_ was the number of plots of each habitat *i*, while *D*_*i*_ and $\bar{L}$ were their width and mean length, respectively.

The availability of urban habitat was estimated by measuring the surface of peripheral urbanized areas (i.e., residential neighborhoods with scattered houses and open gardens where the species can breed, excluding the fully paved core of the city; Fig. 1) in aerial photographs using Q-GIS. We assumed full detectability of breeding owls and nests in this habitat given the high visibility of individuals from the dense street network.

### Breeding habitat selection and density estimation

We assessed whether birds used the different habitat types for breeding in proportion to their availability using the Savage selectivity index *W* = *U*_*i*/_ *p*_*i*_, where *U*_*i*_ is the proportion of breeding pairs recorded in a given habitat and *p*_*i*_ is the proportion of that habitat against total available habitat^74^. The ratio theoretically varies between 0 (full avoidance) and ∞ (full preference). A value of 1 indicates random habitat use, i.e. that the proportion used equals the proportion available^74^. The statistical significance of this index was obtained by comparing the statistic (*wi* − *1*)^*2*^/*se*_*wi*_^*2*^ with the corresponding critical value of a χ^2^ distribution with one degree of freedom, the null hypothesis being that birds use habitats in proportion to their availability. The standard error of the index (*se*_*wi*_) was calculated as √[(*1* − *p*_*i*_)/(*u*_+_ *p_*i*_)], where *u*_+_ is the total number of breeding pairs recorded. Statistical significance was obtained after applying the Bonferroni correction for multiple tests. Breeding density in rural and urban habitats was annually calculated as the number of active nests divided by the measured surface of each habitat.

### Predator surveys

Potential and confirmed predators of burrowing owls include mammals, reptiles and birds. While aerial predators can prey on adults and juveniles when they are in the surrounding of their burrows, terrestrial predators can get into burrows and prey on eggs, chicks and adults. Therefore, we separately considered the abundance and richness of avian and terrestrial predators given their possible differential predation effects.

Relative indexes of richness (number of species) and abundance of predators were obtained by recording all predators observed during a good proportion of our fieldwork sessions, accounting for the time invested on each one to obtain frequencies of observations per hour^75^. Although this method underestimates the abundance of nocturnal predators, the relative indexes obtained are valid for comparing different habitats or sites^75^. Fieldwork sessions usually lasted from one hour before dawn to midday, and from four hours before to one hour after dusk. Species not easily distinguishable in the field such as the large hairy armadillo (*Chaetophractus villosus*) and Southern long-nosed armadillo (*Dasypus hybridus*) were pooled as “armadillos”. Similarly, snakes were pooled for analyses. Domestic dogs were not considered as potential predators because they are fed by their owners and do not represent a true predation threat to birds (authors personal observations).

We employed Generalized Linear Models (GLM) for assessing differences in richness and abundance of predators (response variables; binomial negative error or Poisson error distribution, log link function) between urban and rural habitats (fixed factor), fitting time invested on each fieldwork session as a covariate, and year as a fixed effect.

### Breeding performance

From 2007 to 2014, we repeatedly visited all located nests to record the number of young that reached fledging age. We assessed whether breeding pairs successfully produced at least one fledgling (breeding success), the brood size at fledging of successful pairs (brood size) and the number of young fledged per breeding attempt (productivity). We tried to ascertain the causes of breeding failure, also recording signs of predation such as the presence of corpses or plucked owl feathers at the entrance of the nests.

Differences in breeding parameters between urban and rural nests were assessed through GLMs (breeding success: binomial error distribution, logistic function; (log)brood size: normal error distribution, identity link function; productivity: negative binomial distribution, log-link function). Habitat (urban or rural) and year were fitted as fixed effects, thus controlling for inter-year variability in breeding performance.

### Ethics statements

Field work and procedures were conducted under permits from the Argentinean wildlife agency (22500-4102/09), and the owners of private properties, in accordance with the approved guidelines of the Ethics Committee of CSIC (CEBA-EBD-11-28).

## Electronic supplementary material

Supplementary material
