# Author Correction: Urban conservation hotspots: predation release allows the grassland-specialist burrowing owl to perform better in the city

**DOI:** 10.1038/s41598-023-32913-w

**Published:** 2023-04-12

**Authors:** Natalia Rebolo-Ifrán, José L. Tella, Martina Carrete

**Affiliations:** 1 Departamento de Ecología, Genética y Evolución & IEGEBA-CONICET, Facultad de Ciencias Exactas y Naturales, Universidad de Buenos Aires, Buenos Aires, Argentina; 2 Grupo de Investigadores en Biología de la Conservación (GRINBIC) INIBIOMA-CONICET, Bariloche, Argentina; 3 Department of Conservation Biology, Estación Biológica de Doñana, CSIC, Sevilla, Spain; 4 Department of Physical, Chemical and Natural Systems, Universidad Pablo de Olavide, Sevilla, Spain

Correction to: *Scientific Reports* https://doi.org/10.1038/s41598-017-03853-z, published online 14 June 2017

The original version of this Article contains errors in the Acknowledgements section.

“We thank N. Lois, S. Briones, M. Santillán, P. Laiolo, M. de la Riva, N. Tella-Carrete, and M. Vázquez for their help in capturing and monitoring owls over the years. Field work was conducted under permits from Argentinean wildlife agencies and the owners of private properties.”

should read:

“This work was supported by CGL2015-71378 MINECO/FEDER, UE, Canal Sur TV, Fundación Repsol, Project CGL2012-31888 CGL2015-71378 MITECO, and COOPA20049 from CSIC (Spain). S. Young revised the English. We thank N. Lois, S. Briones, M. Santillán, P. Laiolo, M. de la Riva, N. Tella-Carrete, and M. Vázquez for their help in capturing and monitoring owls over the years. Field work was conducted under permits from Argentinean wildlife agencies and the owners of private properties.”

