# Supplementary material for: Urban conservation hotspots: predation release allows the grassland-specialist burrowing owl to perform better in the city
Source: Sci Rep. 2017 Jun 14;7:3527. doi: 10.1038/s41598-017-03853-z (PMC5471179; doi:10.1038/s41598-017-03853-z)
Supplement: Supplementary file 1 — Supplementary material [file 41598_2017_3853_MOESM1_ESM.pdf]

SUPPLEMENTARY MATERIAL

Urban conservation hotspots: predation release allows the grassland-specialist burrowing owl to perform better in the city

Natalia Rebolo-Ifrán, José L. Tella & Martina Carrete

Fig. S1. Urban and rural habitat availability and their use by burrowing owls in each of the years surveyed. Savage selectivity indexes are shown right to the bars (***: $p < 0.0001$).

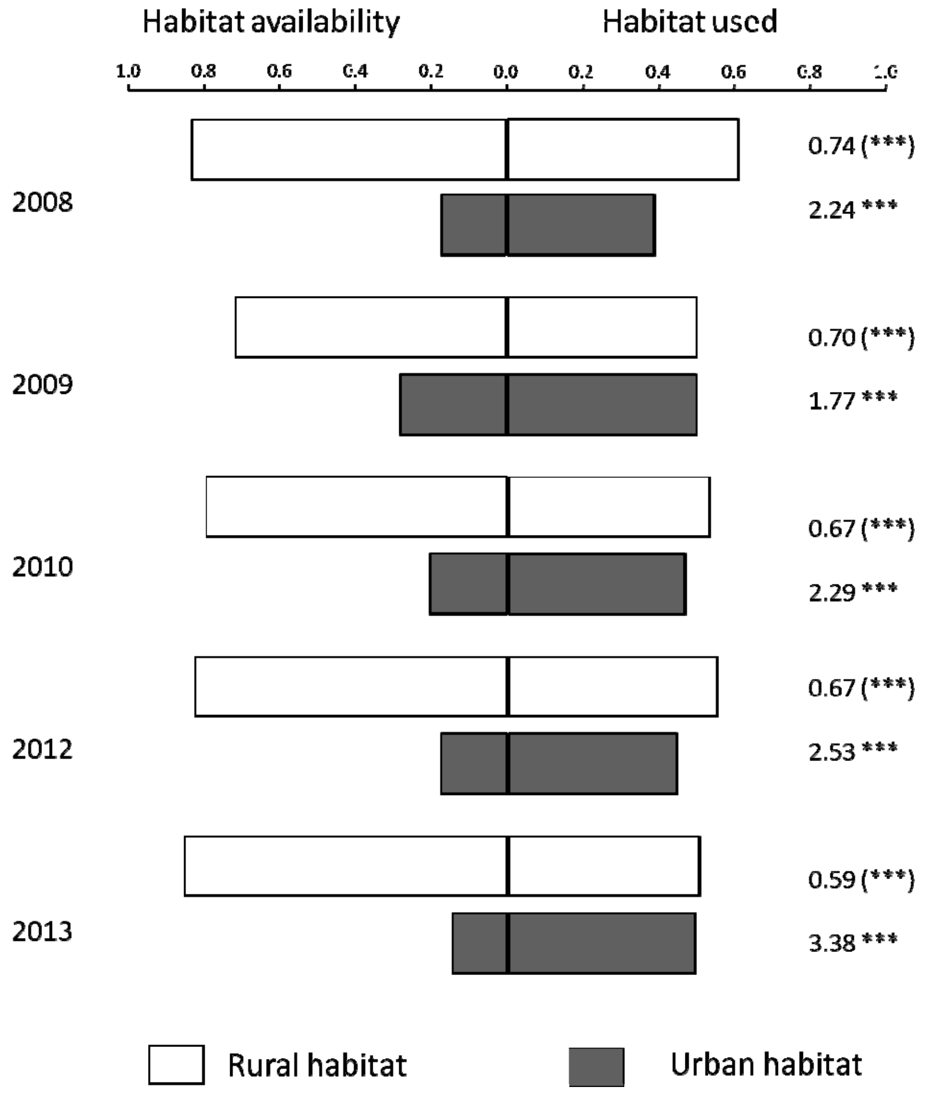

Table S1. Abundance (average ± SE) of aerial and terrestrial predators (individuals/hs) of burrowing owls in urban and rural habitats.

**Avian predators**	**Rural**	**Urban**
Southern Caracara (*Caracara plancus*)	1.30 ± 0.30	0.11 ± 0.04
Long-winged Harrier (*Circus buffoni*)	0.14 ± 0.04	0.02 ± 0.01
Cinereous Harrier (*Circus cinereus*)	0.03 ± 0.01	0.01 ± 0.004
Variable hawk (*Geranoetus polyosoma*)	0.04 ± 0.01	0.003 ± 0.003
Aplomado Falcon (*Falco femoralis*)	0.07 ± 0.01	0.04 ± 0.01
Roadside Hawk (*Rupornis magnirostris*)	0.009 ± 0.004	0.05 ± 0.02
Black-chested Buzzard-eagle (*Geranoaetus melanoleucus*)	0.002 ± 0.002	0 ± 0
Harris's Hawk (*Parabuteo unicinctus*)	0 ± 0	0.02 ± 0.01
**Terrestrial predators**	**Rural**	**Urban**
Domestic cat (*Felis silvestris catus)*	0.02 ± 0.02	0.007 ± 0.003
Pampas Cat (*Leopardus colocolo*)	0.0005 ± 0.005	0 ± 0
Molina's Hog-nosed Skunk (*Conepatus chinga*)	0.015 ± 0.005	0.0007 ± 0.0007
Pampa Fox (*Pseudalopex gymnocercus*)	0.15 ± 0.08	0 ± 0
Lesser grison (*Galictis cuja*)	0.003 ± 0.002	0 ± 0
Armadillos[1]	0.03 ± 0.008	0.006 ± 0.004
Snakes[2]	0.026 ± 0.015	0.009 ±0.008

[1]Large hairy armadillo (*Chaetophractus villosus*) and Southern long-nosed armadillo (*Dasypus hybridus*)

[2]*Paraphimophis (Clelia) rusticus, Philodryas patagoniensis, Erythrolamprus (Liophis) poecilogyrus, Xenodon (Lystrophis) dorbignyi*

Table S2. Models obtained for abundance and richness of potential aerial and terrestrial predators of burrowing owls in urban and rural habitats.

**Abundance of total predators[1]**	**Estimate**	**SE**	**F**	**P**
Habitat (urban)	-1.56	0.12	157.94	< 0.0001
Year			4.19	0.0010
Time	0.07	0.03	6.01	0.0145
**Abundance of aerial predators[1]**	**Estimate**	**SE**	**F**	**P**
Habitat (urban)	-1.50	0.13	131.47	< 0.0001
Year			4.44	0.0006
Time	0.05	0.03	3.29	0.0703
**Abundance of terretrial predators[2]**	**Estimate**	**SE**	χ^2	**P**
Habitat (urban)	-2.02	0.29	77.53	< 0.0001
Year			41.47	< 0.0001
Time	0.17	0.04	17.48	< 0.0001
**Richness of total predators[2]**	**Estimate**	**SE**	χ^2	**P**
Habitat (urban)	-1.09	0.11	117.96	< 0.0001
Year			20.09	0.0012
Time	0.10	0.02	22.24	< 0.0001
**Richness of aerial predators[2]**	**Estimate**	**SE**	χ^2	**P**
Habitat (urban)	-0.95	0.12	74.38	< 0.0001
Year			14.49	0.0128
Time	0.08	0.02	12.96	0.0003
**Richness of terretrial predators[2]**	**Estimate**	**SE**	χ^2	**P**
Habitat (urban)	-1.82	0.31	51.73	< 0.0001
Year			28.97	< 0.0001
Time	0.15	0.05	10.94	0.0009

[1] negative binomial error distribution, log-link function

[2] Poisson error distribution, log-link function
